# Association between insulin resistance, metabolic syndrome and its components and lung cancer: a systematic review and meta-analysis

**DOI:** 10.1186/s13098-024-01308-w

**Published:** 2024-03-11

**Authors:** Jingxuan Liu, Rui Wang, Song Tan, Xiaohu Zhao, Aihua Hou

**Affiliations:** 1 College of Traditional Chinese Medicine, Shandong University of Traditional Chinese Medicine, Jinan, China; 2 Department of Oncology, Yantai Hospital of Traditional Chinese Medicine, Yantai, China

**Keywords:** Lung cancer, Metabolic syndrome, Insulin resistance, Diabetes, Meta-analysis

## Abstract

**Background:**

A growing body of evidence points to the association between insulin resistance (IR), metabolic syndrome (MetS) and its components and lung cancer incidence, but remains controversial and unknown.

**Methods:**

A systematic search was conducted through PubMed, Embase, Cochrane Library, the China National Knowledge Infrastructure (CNKI) and Wanfang databases for the corresponding studies. Each study reported the risk estimate and 95% confidence intervals (CI) for lung cancer, and a fixed effects model or random effects model was used for outcome.

**Results:**

We included 31 publications involving 6,589,383 people with 62,246 cases of lung cancer. Diabetes mellitus (DM) (RR = 1.11, 95% CI  1.06–1.16, *P* = 0.000) and IR (RR = 2.35, 95% CI  1.55–3.58, *P* = 0.000) showed a positive association with lung cancer risk. BMI (RR = 0.66, 95% CI  0.54–0.81, *P* = 0.000) and HDL-C (RR = 0.88, 95% CI  0.79–0.97, *P* = 0.010) were negatively correlated with lung cancer. MetS(RR = 0.99, 95% CI  0.90–1.09, *P* = 0.801), TC (RR = 0.93, 95% CI  0.81–1.06, *P* = 0.274), TG (RR = 0.99, 95% CI  0.88–1.12,*P* = 0.884), LDL-C (RR = 1.01, 95% CI  0.87–1.16, *P* = 0.928), hypertension (RR = 1.01, 95% CI  0.88–1.15, *P* = 0.928), FBG (RR = 1.02, 95% CI  0.92–1.13, *P* = 0.677) and obesity (RR = 1.11, 95% CI  0.92–1.35, *P* = 0.280) were not associated with lung cancer.

**Conclusion:**

Our study showed that the risk of lung cancer is correlated with DM, IR, BMI, and HDL-C. Timely control of these metabolic disorders may have a positive effect on preventing lung cancer.

*Trial registration* Our study has been registered in the Prospective Register of Systematic Reviews (PROSPERO), ID: CRD42023390710.

**Supplementary Information:**

The online version contains supplementary material available at 10.1186/s13098-024-01308-w.

## Introduction

Metabolic syndrome (MetS) is a term that comprises multiple metabolic components, which has a serious impact on health. These metabolic components are mainly obesity, hyperglycaemia, hypertension and dyslipidaemia [1]. Cardiovascular disease and diabetes mellitus (DM), which are most closely related to MetS, are currently the deadliest chronic noncommunicable diseases [2]. Insulin resistance (IR) is the weakening of the responsiveness and sensitivity of tissues to physiological insulin levels, which can lead to metabolic abnormalities and continued progression to type 2 diabetes mellitus (T2DM) and MetS [3].

Lung cancer plays an important role in the global cancer burden with the second highest incidence and the highest mortality [4]. The five-year survival rate is only 22.9% worldwide for lung cancer patients because early clinical symptoms are insidious and when most patients are diagnosed, they have reached the advanced stage [5]. Early diagnosis, screening and identification of risk factors to prevent the disease at its root may go a long way in improving the situation.

Recently, the relationship between MetS and various cancers has been gradually confirmed, which can increase the mortality of cancer patients by 2.4 times [6]. IR is positively correlated with the risk of colorectal cancer [7], prostate cancer [8], endometrial cancer [9], thyroid cancer [10], breast cancer [11] and other cancers. Nevertheless, the relationship between the MetS and its components or IR and lung cancer risk is controversial or unknown. Overall, we tested the hypothesis that MetS, its components and IR are related to lung cancer in this systematic review and meta-analysis.

## Materials and methods

### Methodology and search strategy

This study is based on Preferred Reporting Items for Systematic Reviews and Meta-Analyses (PRISMA) (Additional file 1: Table S1) and is registered in the Prospective Register of Systematic Reviews (PROSPERO), ID: CRD42023390710.

A systematic search was conducted through PubMed, Embase, Cochrane Library, the China National Knowledge Infrastructure (CNKI) and Wanfang databases up to 30 June 2023. The keywords used were “metabolic syndrome”, or “diabetes”, or “insulin resistance”, or “hyperglycemia”, or “hypertension”, or “dyslipidemia”, or “hyperlipidemia”, or “obesity”; and related terms for lung cancer are “lung cancer”, or “pulmonary neoplasm”, or “lung carcinoma”. In addition, the use of “cohort”, or “case”, or “cross-sectional” restricted the search results to cohort studies, case–control studies and cross-sectional studies (Additional file 1: Table S2). Two authors (J.L and R.W) carefully reviewed the references in the articles and hand-searched relevant reviews without time and language restrictions. In case of necessity, we actively contacted the original author to obtain some data. After removing duplicates, two authors (J.L and R.W) independently screened the studies based on title, abstract and complete text. In case of disagreement, we invited the third investigator (X.Z) to discuss and decide.

### Study selection

The included studies must record the risk ratio (RR), odds ratio (OR) or hazard ratio (HR) estimates with 95% confidence intervals (CI) for the incidence of lung cancer, or may be speculated from relevant data. The exposure factors to be analyzed include MetS, its components and IR, such as body mass index (BMI), high-density-lipoprotein cholesterol (HDL-C), total cholesterol (TC), triglyceride (TG), low-density lipoprotein cholesterol (LDL-C), hypertension, DM, obesity and fasting blood glucose (FBG). Normal values or definitions need to be described with similar methods. Studies focused on adults, excluding animal studies and studies of minors. The excluded studies are literature without original data, conference abstracts, case reports, reviews and letters to the editor. We used EndNote version X8.1 (Clarivate Analytics) software to complete the retrieval and preliminary screening.

### Quality assessment

Using the Newcastle–Ottawa Scale (NOS), two authors independently assessed the quality of studies. Outcomes were scored (0–9) by population selection and comparability, including conformity of entry criteria, comparability of research methods and completeness of data. Studies with scores ≥ 5 were recognized as high-quality studies and included in our research.

### Data extraction and analysis

Data were collected from the included literature to extract RR, HR, OR and 95% CI for the relationship between MetS, its components and IR and lung cancer. If studies were multivariate adjusted, multivariate adjusted risk estimates and corresponding 95% CIs were recorded and information on adjusted variables was also recorded. Basic study information (title, authors, year, country, literature source, year of case entry, follow-up time, study design) and case information (number of cases, age, sex, ethnicity) were recorded categorically. The above process was carried out independently by the two researchers, and in case of any disagreement, the disputed article was discussed and reviewed.

We applied random effects models to obtain the total RR and 95% CI. Effect indicators were extracted as effect sizes after adjusting for the most confounding factors if the included studies were corrected for confounding, and raw effect sizes were extracted as study data if the included studies were not corrected for confounding. The results were assessed using RR and 95% CI, and when the effect indicator was HR or OR, it was equated to RR for analysis. Heterogeneity was analysed by the* I*^*2*^ test. When *I*^*2*^ > 50% or *P* of *I*^*2*^ < 0.05, statistical heterogeneity was considered to exist between studies and data were analysed and combined based on a random effects model; when *I*^*2*^ < 50% or *P* of *I*^*2*^ > 0.05, statistical heterogeneity between studies was considered to be low. Sensitivity analysis was used to exclude the data with a large influence in the high heterogeneity group. If no these data were found, we solved heterogeneity with subgroup analysis. For combined results with high heterogeneity in subgroup analyses, regression meta-analysis was performed to identify influencing factors. For the assessment of MetS and its components, the overall effect size and 95% CI were calculated separately for the relevant disorder components. IR was evaluated by the Homeostatic Model Assessment of Insulin Resistance (HOMA-IR), a commonly used surrogate indicator. By sensitivity analysis, we detected the influence of deleting a single document on the total result, and excluded the documents that had a large influence on heterogeneity to ensure the stability of the result. We reported bias evaluation with Begg's test and funnel plots. We performed all statistical analyses using STATA version 17.0 (College Station, TX, USA).

## Results

### Literature search

**Fig. 1:**
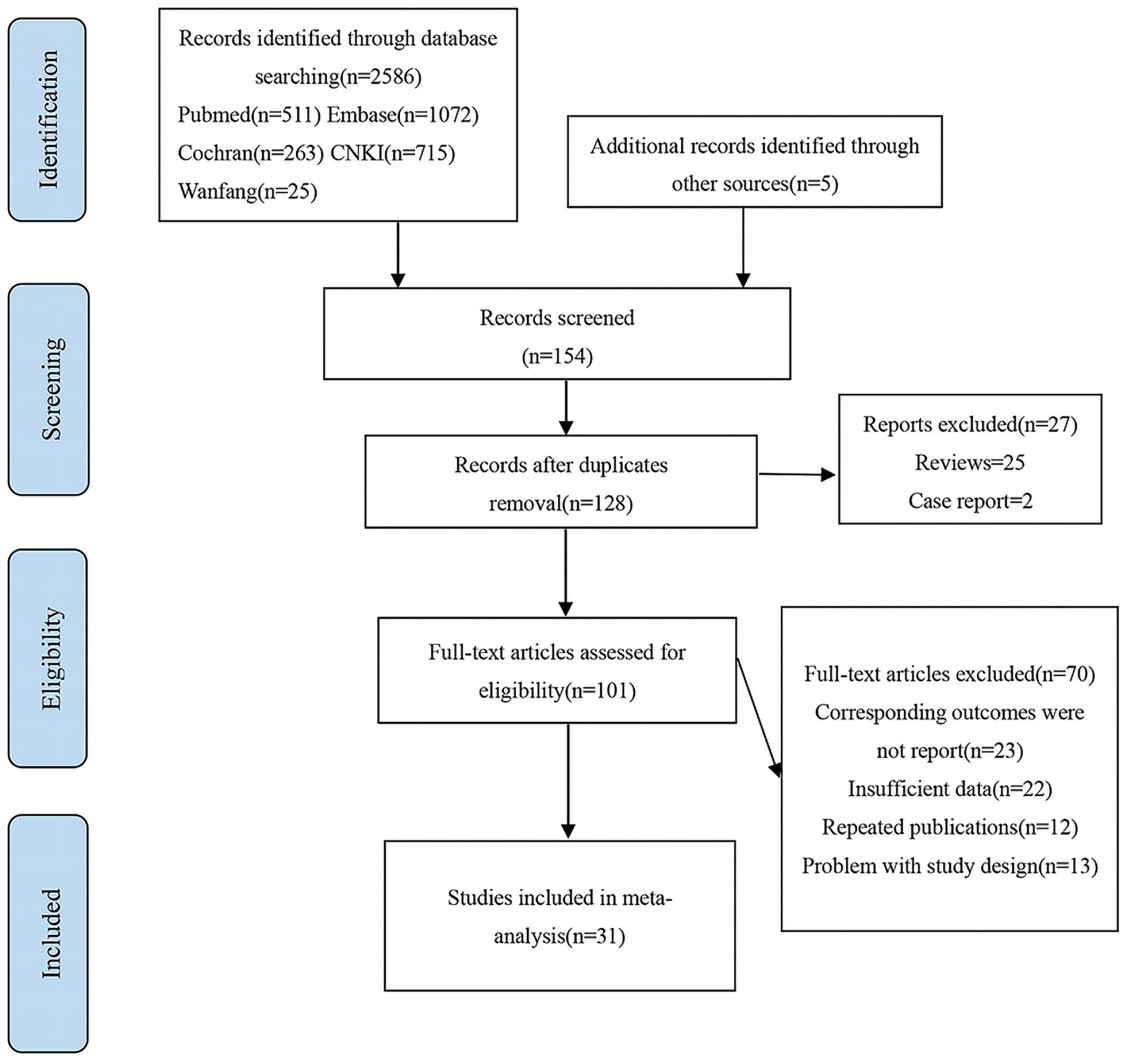
Flow chart of literature screening

A total of 2586 papers were searched from various databases and after careful reading of titles, abstracts, keywords and full texts, 31 papers were finally included in this study. According to statistics, 3 for MetS [12–14],15 for DM [15–28, 30], 11 for lipid indicators [14, 27–29, 31–37], 4 for IR [38–41] and 8 for other associated factors [14, 22, 27, 35, 37–39, 42]. The included literature contained 22 cohort studies and 9 case–control studies. Figure 1 displays a thorough flowchart of the literature screening.

### Literature characteristics

There were 18 Asian studies, 8 European studies, 4 North American studies and 3 Australian studies in the 31 included papers. Of these, two papers reported cases across 3 countries [26, 36]. All the studies we included involved a total of 6,589,383 people, including 62,246 cases of lung cancer. The study that included the largest number of participants amounted to 1,298,385 [22] and a maximum follow-up of 34 years [36]. The mean NOS evaluation score was 7.0, with 87.1% of the studies scoring ≥ 6 and all studies scoring ≥ 5. Most of the literature reported adjusting factors included age, sex, smoking state and family history of malignancy (Additional file 1: Table S3).

### Metabolic disorders and lung cancer

**Fig. 2:**
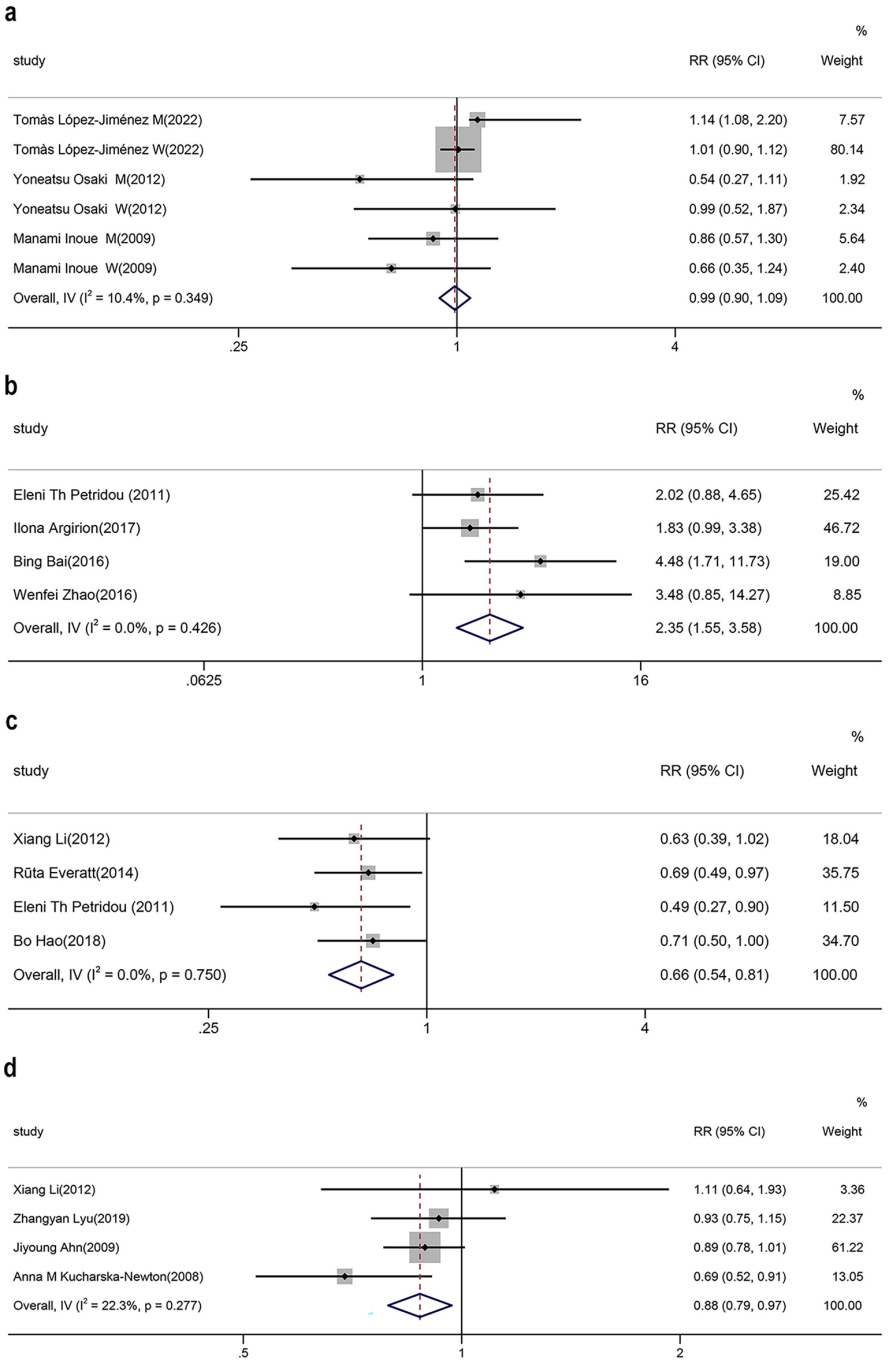
Relationship between metabolic factors and lung cancer. **A** MetS and lung cancer. **B** IR and lung cancer. **C** BMI and lung cancer. **D** HDL-C and lung cancer

**Fig. 3:**
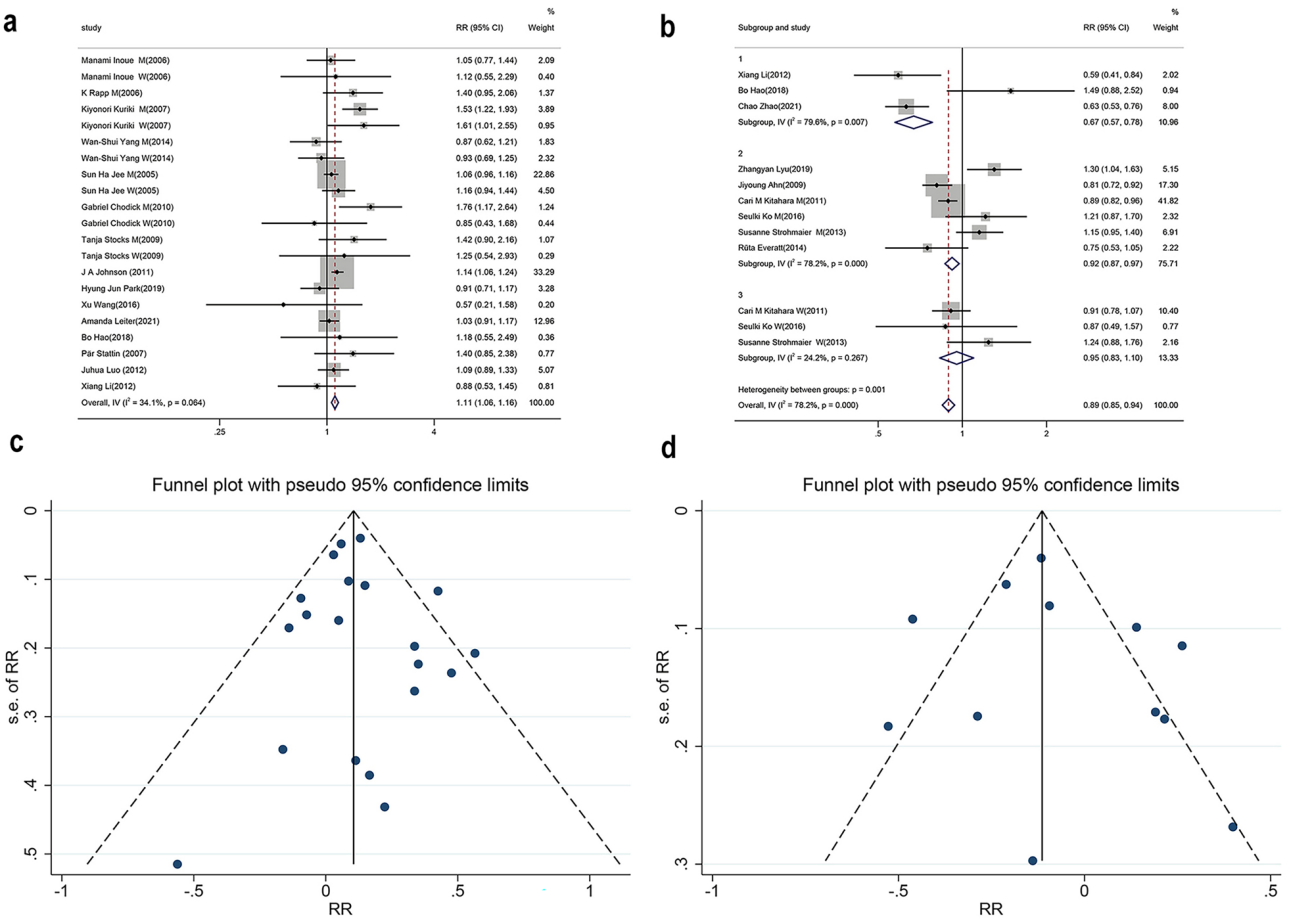
Analysis of DM and TC. **A** Relationship between DM and lung cancer. **B** Relationship between TC and lung cancer (subgroup analysis). **C** Funnel plot of DM. **D** Funnel plot of TC

Analysis actually showed that MetS cannot be proven to increase the risk of lung cancer (RR = 0.99, 95% CI  0.90–1.09, *P* = 0.801, Fig. 2A). In contrast, IR not only promoted the development of lung cancer but showed a higher RR (RR = 2.35, 95% CI  1.55–3.58, *I*^*2*^ = 0.0%, *P* = 0.000, Fig. 2B). As for the components of MetS, DM was positively correlated with lung cancer incidence (RR = 1.11, 95% CI  1.06–1.16, *I*^*2*^ = 34.1%, *P* = 0.000, Fig. 3A). BMI was evidently negative for the risk of lung cancer (RR = 0.66, 95% CI  0.54–0.81,* I*^*2*^ = 0.0%, *P* = 0.000, Fig. 2C). In comparison, TG (RR = 0.99, 95% CI  0.88–1.12, *I*^*2*^ = 29.4%, *P* = 0.884, Additional file 1: Fig. S1A), LDL-C (RR = 1.01, 95% CI  0.87–1.16, *I*^*2*^ = 45.5%, *P* = 0.928, Additional file 1: Fig. S1B), hypertension (RR = 1.01, 95% CI  0.88–1.15, *I*^*2*^ = 0.0%, *P* = 0.928, Additional file 1: Fig. S1C), FBG (RR = 1.02, 95% CI  0.92–1.13, *I*^*2*^ = 16.1%, *P* = 0.677, Additional file 1: Fig. S1D), and obesity (RR = 1.11, 95% CI  0.92–1.35, *I*^*2*^ = 0.0%, *P* = 0.280, Additional file 1: Fig. S1E) were not associated with lung cancer development. (Table 1, Fig. 2).

**Table 1:** Results of meta-analysis (after including sensitivity analysis and subgroup analysis)

Factors	Datasets	RR (95% CI)	P	I^2^ (%)	P of I^2^	Model	P of Begg’s test
MetS	6	0.99(0.90–1.09)	0.801	10.4	0.349	F	0.060
BMI	4	0.66(0.54–0.81)	0.000	0.0	0.750	F	0.308
HDL-C	5	0.73(0.54–0.99)	0.041	83.4	0.000	R	–
HDL-C^a^	4	0.88(0.79–0.97)	0.010	22.3	0.277	F	0734
TG	6	0.99(0.88–1.12)	0.884	29.4	0.215	F	0.452
TC	12	0.93(0.81–1.06)	0.274	78.2	0.000	R	0.732
Both sex	3	0.77(0.50–1.19)	0.246	79.6	0.007	R	–
Men	6	0.99(0.84–1.15)	0.852	78.2	0.000	R	–
Women	3	0.98(0.80–1.19)	0.827	24.2	0.267	R	–
LDL-C	4	1.01(0.87–1.16)	0.928	45.5	0.139	F	0.734
Hypertension	6	1.01(0.88–1.15)	0.928	0.0	0.794	F	1.000
FBG	5	1.02(0.92–1.13)	0.677	16.1	0.312	F	0.462
DM	21	1.11(1.06–1.16)	0.000	34.1	0.064	F	0.833
Obesity	5	1.11(0.92–1.35)	0.280	0.0	0.445	F	0.462
IR	4	2.35(1.55–3.58)	0.000	0.0	0.426	F	0.308

MetS: metabolic syndrome, FBG: fasting blood glucose, TG: triglyceride, TC: total cholesterol, HDL-C: high-density lipid-cholesterol, LDL-C: low-density lipoprotein cholesterol, BMI: body mass index, DM: diabetes mellitus, IR: insulin resistance, F: Fixed effects model, R: Random effects model

^a^By sensitivity analysis

High heterogeneity was seen in the HDL-C (*I*^*2*^ = 83.4%) and TC (*I*^*2*^ = 78.2%) data. We performed sensitivity analysis and revealed a large effect of Hao’s data in the HDL-C group. Exclusion of these data resulted in significantly lower heterogeneity (*I*^*2*^ = 22.3%), also demonstrating a negative correlation between HDL-C and the incidence of lung cancer (RR = 0.88, 95% CI  0.79–0.97, *I*^*2*^ = 22.3%, *P* = 0.010, Fig. 2D).

The TC group’s sensitivity analysis showed that no data were found for a significant effect on lung cancer incidence. Subgroup analysis according to sex revealed reduced heterogeneity of TC indicators in women and no connection with lung cancer incidence (RR = 0.98, 95% CI  0.80–1.19, *I*^*2*^ = 24.2%,* P* = 0.827, Fig. 3B). Nonetheless, there was still significant heterogeneity in groups of both sex (*I*^*2*^ = 79.6%) and men (*I*^*2*^ = 78.2). We performed meta-regressions for factors that may have influenced (study type, geography, age), but negative results emerged (Adj R-squared = 33.20%, I-squared_res = 69.56%, F = 0.3402). Because of the apparent variability in the relevant information recorded across studies, we ultimately found no additional factors that might have influenced heterogeneity. Our study was unable to demonstrate a correlation between TC and lung cancer incidence.

### Publication bias

**Fig. 4:**
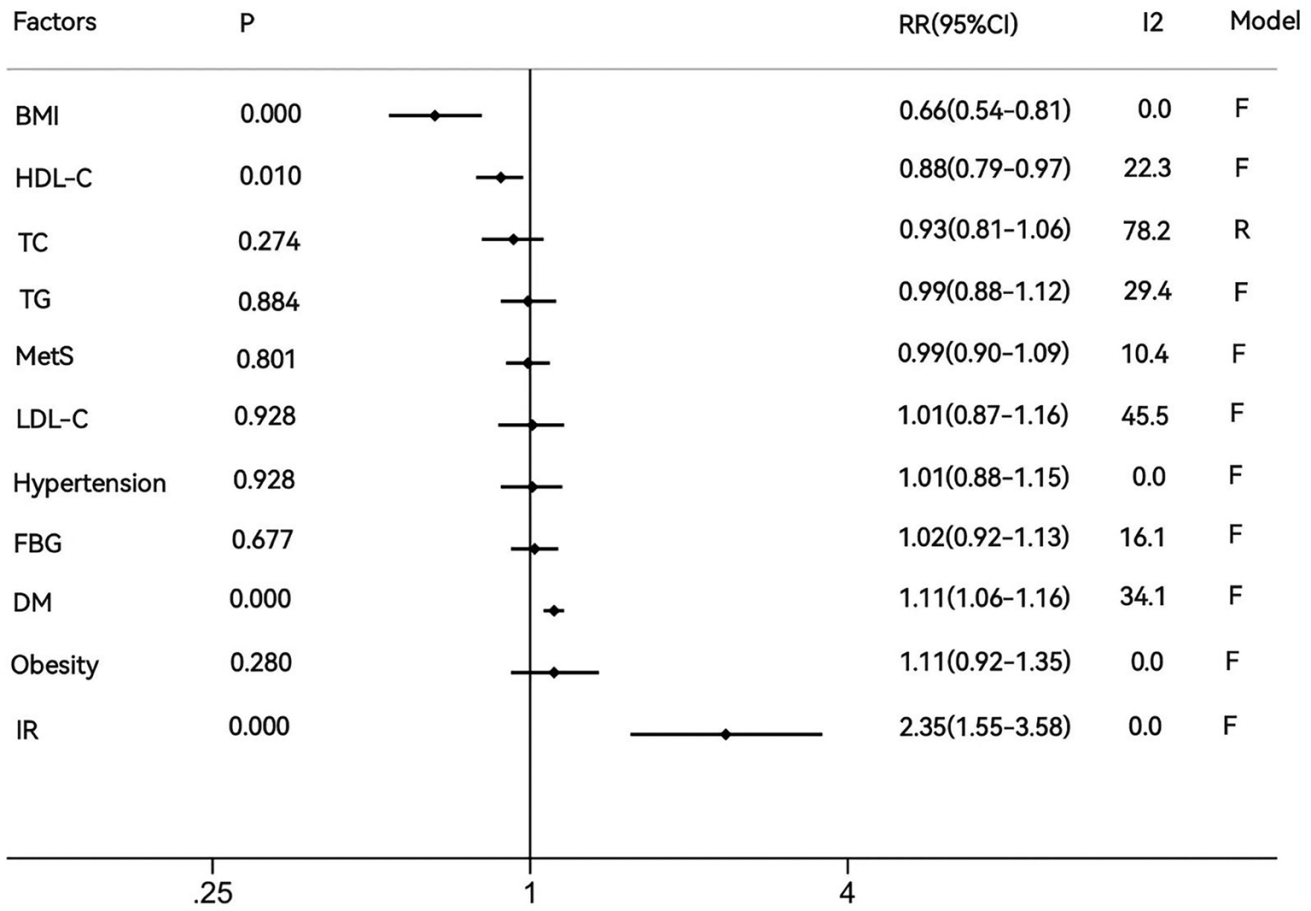
Summary data

The funnel plots for all metabolic factors showed basic symmetry, with those for the DM and TC groups shown below (Fig. 3C, D). No publication bias was found in Begg’s test (*P* > 0.05). A summary graph of all results is displayed in Fig. 4.

## Discussion

The aim of this study was to explore the relationship between MetS, its components and IR and lung cancer. The most important result of this work is that the MetS cannot be summarized as a risk factor influencing the development of lung cancer. However, among the components of the MetS, DM was positively associated with lung cancer risk, and BMI and HDL-C were negatively associated with lung cancer risk. Interestingly, IR, which is closely related to the MetS, showed a strong correlation with lung cancer risk.

In recent years, there has been growing epidemiological evidence that MetS is associated with the occurrence or progression of a variety of cancers [43–47]. Our statistical findings are not sufficient to demonstrate a positive association between lung cancer and MetS, a conclusion that is not one-sided. MetS appears to be more closely associated with a higher incidence of colorectal cancer in both sexes [48], liver cancer in men [49], and breast cancer in women [50], as evidenced by the large body of literature on targeted studies of MetS and the above cancers. The current mechanisms linking MetS to cancer risk are not well defined. We investigated the relationship between lung cancer and metabolic components of MetS as much as possible to provide direction to other researchers in their search for specific mechanisms.

DM is the most represented disorder of glucose metabolism. Our findings suggest that there is a connection between DM and lung cancer, which is consistent with other literature [51–53]. In a 2013 meta-analysis, Lee et al. suggested that pre-existing DM may promote the occurrence of lung cancer, especially in women with DM. However, this association disappeared when the confounding factor of smoking was not controlled [51]. A subsequent, more comprehensive meta-analysis also supported the connection between DM and lung cancer in women, suggesting that the effect of DM on lung cancer risk may be influenced by smoking status. This could explain why the majority of research found a weak correlation between lung cancer in men with DM [53]. A prospective cohort study from the United States, the first to examine overall survival in women with DM, suggests that women with lung cancer who have pre-existing DM had a worse overall survival rate [54]. The exact mechanism by which DM affects the development of lung cancer is unclear, and epithelial-to-mesenchymal transition (EMT) pathway-mediated lung fibrosis under activation of the inflammatory factor transforming growth factor beta1(TGF-β1) [55] with high insulin like growth factor 1 receptor (IGF-1R) and insulin receptor substrate 2 (IRS-2) protein expression [56] may be a potential factor.

The evidence we have collected so far does not support a relationship between high FBG and lung cancer, and the information we have gathered thus far does not support such an association either [22, 35, 39]. The results of Huang et al. show that abnormal fasting plasma glucose (FPG) (FPG between 5.6–6.9 mmol/l and 6.1–6.9 mmol/l) is associated with an increased overall cancer risk, but site specific secondary analyses also found it to be unrelated to lung cancer [57]. However, their study excluded individuals with FPG ≥ 7.1 mmol/l, a slight difference from our criteria. In addition, either too high (> 110 mg/dl) [58] or too low (< 91 mg/dl) FBG reduced survival in patients with NSCLC [59].

Dysregulation of lipid metabolism is a prominent metabolic manifestation of cancer, and cancer cells promote their own proliferation, survival, migration, invasion and metastasis through the regulation of lipid metabolism [60]. Our analysis showed that BMI was negatively correlated with lung cancer but obesity was not. Petrelli et al. found that obesity was associated with increased overall cancer mortality, but for lung cancer patients, those with concurrent obesity had significantly longer survival than those without obesity [61]. According to several studies, BMI is inversely related to lung cancer risk [62], which is consistent with our results. However, this relationship disappears when discussing only BMI in nonsmoking lung cancer patients [63]. Therefore, even after adjustment, our results may still be confounded by smoking. In response to this controversy, Zhu et al. published a meta-analysis of statistics with 29 observational studies of never-smokers. Their results still suggest that a greater BMI is linked to a lower risk of lung cancer, particularly in women [64]. It should be noted that the “obesity paradox” exists in some chronic diseases. In some studies of pulmonary [65] or cardiovascular disease [66], obese people have a longer expected survival time, but this does not mean that obesity is a protective factor for these diseases. The obese group may represent people who have not lost weight and muscle mass as a result of the disease and who have higher nutritional reserves in their bodies, which has been suggested by some researchers as the reason for the existence of the “obesity paradox”. The BMI shown in this study does not fully represent excess body fat, which may also be the reason for presenting a negative correlation between BMI and lung cancer risk. The exact reasons for this inverse relationship remain to be investigated, and this result still needs to be considered with caution.

Hypertension and cancer-specific mortality had a positive connection [67]. A recent study on the prevalence of comorbidities in a high-risk group for lung cancer showed that hypertension was the most significant comorbidity (approximately 35.2%) [68]. Zeng et al. examined 181 patients with advanced NSCLC with T2DM and suggested that hypertension may worsen their prognosis [69]. Among patients with lung cancer, another related study also suggests that hypertension is not a risk factor for postoperative readmission [70]. The differences in these results may be influenced by sample size, age of participants and the use of antihypertensive drugs. There is currently no solid proof that hypertension and lung cancer are related.

In our analysis of the connection between the lipid profile and lung cancer, we found that low HDL-C will increase the incidence of lung cancer, although TG, TC, and LDL-C had no concern with it. Lin's analysis differed slightly from our results in that their data showed a positive association between TG (RR = 1.68, 95% CI  1.44–1.96) and lung cancer risk, and an inverse association in TC group, whose findings for HDL-C analysis (RR = 0.76, 95% CI  0.59–0.97) were consistent with ours [71]. The studies we analysed for TG were all Asian, where TG levels are inherently slightly higher compared to Caucasians [72], so there may be unavoidable confounding in the data. The connection between TC and lung cancer development has received less attention and is unclear. In vitro studies have shown that lung adenocarcinoma cells are encouraged to migrate and invade by 25-hydroxycholesterol [73]. Our study could not prove the connection between high TC and lung cancer risk, but the exact mechanism remains to be elucidated. Zhou et al. clearly suggested that high expression of HDL-C reduces the risk of death in lung cancer patients [74], which may be associated with the anti-inflammatory and antioxidant activities of HDL that inhibit tumor cell proliferation [75, 76]. This finding was also confirmed in the study by Hao et al. that the risk of lung cancer is lower and the survival of lung cancer patients is higher with higher HDL-C levels [77]. In addition, low HDL-C is one of the hallmarks of IR. However, the condition of patients with low HDL-C in the studies we included was limited to the value of the index, and it was not documented in detail whether these patients were also IR patients. So the effect of IR on the results in the HDL-C group is questionable. We believe that the confounding of the HDL-C group by IR is limited. Because IR is often accompanied by disorganization of multiple factors. The studies which we included were adjusted for the relevant factors. For LDL-C, the corresponding epidemiological data are imperfect and we only included four case–control studies from China. More researches are necessary to explore the impact of LDL-C levels on lung cancer.

The relevance of IR to cancer has been demonstrated in clinical studies. Insulin, as a peptide hormone that stimulates tissue accretion, has a cancer-promoting effect [78, 79]. Karlstad et al. demonstrated that insulin use increases the risk of lung cancer [80]. Hyperinsulinemia can contribute to increased cancer incidence [81] and mortality [82]. At the same time, MetS, which is closely related to IR, was not identified in 2 meta-analyses as a factor promoting the development of lung cancer [83, 84]. However, the triglyceride glucose index (TyG), a more convenient index of IR, was proven to be related to cancer risk (RR = 1.14, 95% CI  1.08–1.20, *P* < 0.001) [85]. Our study found a high positive association between IR and lung cancer risk in 463 lung cancer patients out of 1,175 participants. Furthermore, there is currently insufficient epidemiological evidence connecting IR with lung cancer. However, there are only four included case–control studies, in which small samples and unclear adjustment factors may confuse the results.

## Limitations

Our study provides a comprehensive analysis of the influence of MetS, IR and related factors on the development of lung cancer and compares the results of different factors. This is the first analysis of hypertension, FBG and IR, but there was unexplained heterogeneity in the TC subgroup across both sex and men, which may reduce the reliability of the results. In addition, some of the factors were less well studied and more consistent results might have been obtained if more studies could have been included.

## Conclusion

DM and IR are expected to increase lung cancer risk, especially IR. Meanwhile, there was a negative correlation between BMI and HDL-C and lung cancer. MetS, TC, TG, LDL-C, hypertension, obesity and FBG are not associated with the development of lung cancer. These results might indicate that controlling the condition of DM and IR patients in time and improving the physical condition of patients with low BMI or low HDL-C have a positive effect on preventing lung cancer. Further clinical studies and mechanistic studies are needed to clarify the relationship of MetS, its components and IR with lung cancer risk.

### Supplementary Information

**Additional file 1: Table S1.** PRISMA checklist. **Table S2.** PubMed retrieval strategy. **Table S3.** Literature characteristics. **Figure S1.** Analysis figure of metabolic factors unrelated to lung cancer. **A** TG. **B** LDL-C. **C** Hypertension. **D** FBG. **E** Obesity.

## Data Availability

All data generated or analysed during this study are included in this published article and its Additional files.

## Abbreviations

BMI: Body mass index
CI: Confidence interval
DM: Diabetes mellitus
FBG: Fasting blood glucose
FPG: Fasting plasma glucose
HDL-C: High-density-lipoprotein cholesterol
HR: Hazard ratio
HOMA-IR: Homeostatic Model Assessment of Insulin Resistance
IR: Insulin resistance
LDL-C: Low-density lipoprotein cholesterol
MetS: Metabolic syndrome
NOS: Newcastle–Ottawa Scale
NSCLC: Non-small cell lung cancer
OR: Odds ratio
RR: Risk ratio
TC: Total cholesterol
TG: Triglyceride
T2DM: Type 2 diabetes mellitus

## References

[1] Alberti KG, Eckel RH, Grundy SM (2009). Harmonizing the metabolic syndrome: a joint interim statement of the International Diabetes Federation Task Force on Epidemiology and Prevention; National Heart, Lung, and Blood Institute; American Heart Association; World Heart Federation; International Atherosclerosis Society; and International Association for the Study of Obesity. Circulation.

[2] NCD Countdown 2030 collaborators (2018). NCD Countdown 2030: worldwide trends in non-communicable disease mortality and progress towards Sustainable Development Goal target 3.4. Lancet..

[3] Freeman AM, Pennings N, Freeman AM (2022). Insulin resistance. StatPearls.

[4] Sung H, Ferlay J, Siegel RL (2021). Global cancer statistics 2020: GLOBOCAN estimates of incidence and mortality worldwide for 36 cancers in 185 countries. CA Cancer J Clin.

[5] Wood DE, Kazerooni EA, Aberle D (2022). NCCN guidelines insights: lung cancer screening, version 1.2022. J Natl Compr Canc Netw..

[6] Scherübl H (2022). Metabolic syndrome and cancer risk. Dtsch Med Wochenschr.

[7] Xu J, Ye Y, Wu H (2016). Association between markers of glucose metabolism and risk of colorectal cancer. BMJ Open..

[8] Saboori S, Rad EY, Birjandi M, Mohiti S, Falahi E (2019). Serum insulin level, HOMA-IR and prostate cancer risk: a systematic review and meta-analysis. Diabetes Metab Syndr.

[9] Hernandez AV, Pasupuleti V, Benites-Zapata VA, Thota P, Deshpande A, Perez-Lopez FR (2015). Insulin resistance and endometrial cancer risk: a systematic review and meta-analysis. Eur J Cancer.

[10] Zhao J, Zhang Q, Yang Y, Yao J, Liao L, Dong J (2021). High prevalence of thyroid carcinoma in patients with insulin resistance: a meta-analysis of case-control studies. Aging (Albany NY).

[11] Hernandez AV, Guarnizo M, Miranda Y (2014). Association between insulin resistance and breast carcinoma: a systematic review and meta-analysis. PLoS ONE.

[12] López-Jiménez T, Duarte-Salles T, Plana-Ripoll O, Recalde M, Xavier-Cos F, Puente D (2022). Association between metabolic syndrome and 13 types of cancer in Catalonia: a matched case-control study. PLoS ONE..

[13] Osaki Y, Taniguchi S, Tahara A, Okamoto M, Kishimoto T (2012). Metabolic syndrome and incidence of liver and breast cancers in Japan. Cancer Epidemiol.

[14] Inoue M, Noda M, Kurahashi N (2009). Impact of metabolic factors on subsequent cancer risk: results from a large-scale population-based cohort study in Japan. Eur J Cancer Prev.

[15] Rapp K, Schroeder J, Klenk J (2006). Fasting blood glucose and cancer risk in a cohort of more than 140,000 adults in Austria. Diabetologia.

[16] Kuriki K, Hirose K, Tajima K (2007). Diabetes and cancer risk for all and specific sites among Japanese men and women. Eur J Cancer Prev.

[17] Johnson JA, Bowker SL, Richardson K, Marra CA (2011). Time-varying incidence of cancer after the onset of type 2 diabetes: evidence of potential detection bias. Diabetologia.

[18] Park HJ, Joh HK, Choi S, Park SM (2019). Type 2 diabetes mellitus does not increase the risk of lung cancer among never-smokers: a nationwide cohort study. Transl Lung Cancer Res.

[19] Yang WS, Yang Y, Yang G (2014). Pre-existing type 2 diabetes and risk of lung cancer: a report from two prospective cohort studies of 133,024 Chinese adults in urban Shanghai. BMJ Open..

[20] Wang X, Li QL, Liu Y (2016). Correlation between type 2 diabetes mellitus and lung cancer. Oncol Prog..

[21] Leiter A, Charokopos A, Bailey S (2021). Assessing the association of diabetes with lung cancer risk. Transl Lung Cancer Res.

[22] Jee SH, Ohrr H, Sull JW, Yun JE, Ji M, Samet JM (2005). Fasting serum glucose level and cancer risk in Korean men and women. JAMA.

[23] Stattin P, Björ O, Ferrari P (2007). Prospective study of hyperglycemia and cancer risk. Diabetes Care.

[24] Luo J, Chlebowski R, Wactawski-Wende J, Schlecht NF, Tinker L, Margolis KL (2012). Diabetes and lung cancer among postmenopausal women. Diabetes Care.

[25] Chodick G, Heymann AD, Rosenmann L (2010). Diabetes and risk of incident cancer: a large population-based cohort study in Israel. Cancer Causes Control.

[26] Stocks T, Rapp K, Bjørge T (2009). Blood glucose and risk of incident and fatal cancer in the metabolic syndrome and cancer project (me-can): analysis of six prospective cohorts. PLoS Med.

[27] Hao B, Yu M, Sang C, Bi B, Chen J (2018). Dyslipidemia and non-small cell lung cancer risk in Chinese population: a case-control study. Lipids Health Dis..

[28] Li X. Related factors of type 2 diabetes mellitus patients with lung cancer. Guangxi Medical University. 2012. https://kns.cnki.net/kcms/detail/detail.aspx?FileName=1012349108.nh&DbName=CMFD2012 (In Chinese). Accessed 14 July 2023

[29] Lyu Z, Li N, Wang G (2019). Independent and joint associations of blood lipids and lipoproteins with lung cancer risk in Chinese males: a prospective cohort study. Int J Cancer.

[30] Inoue M, Iwasaki M, Otani T, Sasazuki S, Noda M, Tsugane S (2006). Diabetes mellitus and the risk of cancer: results from a large-scale population-based cohort study in Japan. Arch Intern Med.

[31] Ahn J, Lim U, Weinstein SJ (2009). Prediagnostic total and high-density lipoprotein cholesterol and risk of cancer. Cancer Epidemiol Biomarkers Prev.

[32] Kitahara CM, Berrington de González A, Freedman ND (2011). Total cholesterol and cancer risk in a large prospective study in Korea. J Clin Oncol..

[33] Kucharska-Newton AM, Rosamond WD, Schroeder JC (2008). HDL-cholesterol and the incidence of lung cancer in the Atherosclerosis Risk in Communities (ARIC) study. Lung Cancer.

[34] Zhao C (2021). Independent associations between blood lipid profiles and lung cancer risk. Shandong Univ.

[35] Ko S, Yoon SJ, Kim D, Kim AR, Kim EJ, Seo HY (2016). Metabolic risk profile and cancer in Korean men and women. J Prev Med Public Health.

[36] Strohmaier S, Edlinger M, Manjer J (2013). Total serum cholesterol and cancer incidence in the metabolic syndrome and cancer project (Me-Can). PLoS ONE.

[37] Everatt R, Virvičiūtė D, Kuzmickienė I, Tamošiūnas A (2014). Body mass index, cholesterol level and risk of lung cancer in Lithuanian men. Lung Cancer.

[38] Petridou ET, Sergentanis TN, Antonopoulos CN (2011). Insulin resistance: an independent risk factor for lung cancer?. Metabolism.

[39] Argirion I, Weinstein SJ, Männistö S, Albanes D, Mondul AM (2017). Serum insulin, glucose, indices of insulin resistance, and risk of lung cancer. Cancer Epidemiol Biomarkers Prev.

[40] Bai B. Blood glucose and insulin resistance in lung cancer patients. Zhengzhou University. 2016. https://d.wanfangdata.com.cn/thesis/ChJUaGVzaXNOZXdTMjAyMzAxMTISB0Q4Mzk1MjIaCGFhc2RlNGd4 (In Chinese). Accessed 15 July 2023

[41] Zhao WF. The level and significance of insulin resistance in patients with lung cancer. Zhengzhou University. 2016. https://kns.cnki.net/KCMS/detail/detail.aspx?dbname=CMFD201701&filename=1016175784.nh. (In Chinese). Accessed 15 July 2023

[42] Tseng CH (2014). Diabetes but not insulin increases the risk of lung cancer: a Taiwanese population-based study. PLoS ONE..

[43] Chung KC, Juang SE, Chen HH (2022). Association between metabolic syndrome and colorectal cancer incidence and all-cause mortality: a hospital-based observational study. BMC Gastroenterol..

[44] Hernández-Pérez JG, Torres-Sánchez L, Hernández-Alcaráz C (2022). Metabolic syndrome and prostate cancer risk: a population case-control study. Arch Med Res.

[45] Palmiero P, Maiello M, Cecere A, Ciccone MM (2021). Metabolic syndrome and breast cancer: a dangerous association for postmenopausal women. Acta Biomed..

[46] Song JL, Li LR, Yu XZ (2022). Association between metabolic syndrome and clinicopathological features of papillary thyroid cancer. Endocrine.

[47] Park B (2022). Associations between obesity, metabolic syndrome, and endometrial cancer risk in East Asian women. J Gynecol Oncol.

[48] Esposito K, Chiodini P, Capuano A (2013). Colorectal cancer association with metabolic syndrome and its components: a systematic review with meta-analysis. Endocrine.

[49] Yu MW, Lin CL, Liu CJ, Yang SH, Tseng YL, Wu CF (2017). Influence of metabolic risk factors on risk of hepatocellular carcinoma and liver-related death in men with chronic Hepatitis B: a large cohort study. Gastroenterology.

[50] Kabat GC, Kim M, Chlebowski RT (2009). A longitudinal study of the metabolic syndrome and risk of postmenopausal breast cancer. Cancer Epidemiol Biomarkers Prev.

[51] Lee JY, Jeon I, Lee JM, Yoon JM, Park SM (2013). Diabetes mellitus as an independent risk factor for lung cancer: a meta-analysis of observational studies. Eur J Cancer.

[52] Sona MF, Myung SK, Park K, Jargalsaikhan G (2018). Type 1 diabetes mellitus and risk of cancer: a meta-analysis of observational studies. Jpn J Clin Oncol.

[53] Yi ZH, Luther Y, Xiong GH (2020). Association between diabetes mellitus and lung cancer: meta-analysis. Eur J Clin Invest.

[54] Luo J, Hendryx M, Qi L, Ho GY, Margolis KL (2016). Pre-existing diabetes and lung cancer prognosis. Br J Cancer.

[55] Talakatta G, Sarikhani M, Muhamed J (2018). Diabetes induces fibrotic changes in the lung through the activation of TGF-β signaling pathways. Sci Rep..

[56] Ding J, Tang J, Chen X (2013). Expression characteristics of proteins of the insulin-like growth factor axis in non-small cell lung cancer patients with preexisting type 2 diabetes mellitus. Asian Pac J Cancer Prev.

[57] Huang Y, Cai X, Qiu M (2014). Prediabetes and the risk of cancer: a meta-analysis. Diabetologia.

[58] Kirakli EK, Yilmaz U, Yilmaz H, Komurcuoglu B (2018). Fasting blood glucose level in locally advanced non-small cell lung cancer: a new prognostic factor?. Horm Cancer.

[59] Yang JR, Chen GC, Xu JY (2019). Fasting blood glucose levels and prognosis in patients with non-small-cell lung cancer: a prospective cohort study in China. Onco Targets Ther..

[60] Bian X, Liu R, Meng Y, Xing D, Xu D, Lu Z (2021). Lipid metabolism and cancer. J Exp Med.

[61] Petrelli F, Cortellini A, Indini A (2021). Association of obesity with survival outcomes in patients with cancer: a systematic review and meta-analysis. JAMA Netw Open..

[62] Yang Y, Dong J, Sun K (2013). Obesity and incidence of lung cancer: a meta-analysis. Int J Cancer.

[63] Duan P, Hu C, Quan C (2015). Body mass index and risk of lung cancer: systematic review and dose-response meta-analysis. Sci Rep..

[64] Zhu H, Zhang S (2018). Body mass index and lung cancer risk in never smokers: a meta-analysis. BMC Cancer..

[65] Li S, Wang Z, Huang J, Fan J, Du H, Liu L, Che G (2017). Systematic review of prognostic roles of body mass index for patients undergoing lung cancer surgery: does the 'obesity paradox' really exist?. Eur J Cardiothorac Surg.

[66] Hastie CE, Padmanabhan S, Slack R (2010). Obesity paradox in a cohort of 4880 consecutive patients undergoing percutaneous coronary intervention. Eur Heart J.

[67] Petrelli F, Ghidini A, Cabiddu M (2021). Effects of hypertension on cancer survival: a meta-analysis. Eur J Clin Invest.

[68] Almatrafi A, Thomas O, Callister M, Gabe R, Beeken RJ, Neal R (2023). The prevalence of comorbidity in the lung cancer screening population: a systematic review and meta-analysis. J Med Screen.

[69] Zeng X, Zeng D, Cheng J (2020). Influence of hypertension on the survival of non-small cell lung cancer patients with type 2 diabetes mellitus. Med Sci Monit..

[70] Liu J, Yang X, Liu X, Xu Y, Huang H (2022). Predictors of readmission after pulmonary resection in patients with lung cancer: a systematic review and meta-analysis. Technol Cancer Res Treat.

[71] Lin X, Lu L, Liu L (2017). Blood lipids profile and lung cancer risk in a meta-analysis of prospective cohort studies. J Clin Lipidol.

[72] Gijsberts CM, den Ruijter HM, Asselbergs FW, Chan MY, de Kleijn DP, Hoefer IE (2015). Biomarkers of coronary artery disease differ between Asians and Caucasians in the general population. Glob Heart.

[73] Chen L, Zhang L, Xian G, Lv Y, Lin Y, Wang Y (2017). 25-Hydroxycholesterol promotes migration and invasion of lung adenocarcinoma cells. Biochem Biophys Res Commun..

[74] Zhou P, Li B, Liu B, Chen T, Xiao J (2018). Prognostic role of serum total cholesterol and high-density lipoprotein cholesterol in cancer survivors: a systematic review and meta-analysis. Clin Chim Acta.

[75] Su F, Grijalva V, Navab K (2012). HDL mimetics inhibit tumor development in both induced and spontaneous mouse models of colon cancer. Mol Cancer Ther.

[76] Ruscica M, Botta M, Ferri N (2018). High density lipoproteins inhibit oxidative stress-induced prostate cancer cell proliferation. Sci Rep..

[77] Hao B, Bi B, Sang C (2019). Systematic review and meta-analysis of the prognostic value of serum high-density lipoprotein cholesterol levels for solid tumors. Nutr Cancer.

[78] Maghlaperidze Z, Kapetivadze V, Tabukashvili R, Lazashvili T, Kuparadze M, Gratiashvili E (2021). The role of insulin-like growth factor-1 and insulin in development of colorectal cancer. Georgian Med News.

[79] Saisana M, Griffin SM, May FEB (2022). Insulin and the insulin receptor collaborate to promote human gastric cancer. Gastric Cancer.

[80] Karlstad O, Starup-Linde J, Vestergaard P (2013). Use of insulin and insulin analogs and risk of cancer: systematic review and meta-analysis of observational studies. Curr Drug Saf.

[81] Loftfield E, Freedman ND, Lai GY (2016). Higher glucose and insulin levels are associated with risk of liver cancer and chronic liver disease mortality among men without a history of diabetes. Cancer Prev Res (Phila).

[82] Kira S, Ito C, Fujikawa R, Misumi M (2020). Increased cancer mortality among Japanese individuals with hyperinsulinemia. Metabol Open..

[83] Esposito K, Chiodini P, Colao A, Lenzi A, Giugliano D (2012). Metabolic syndrome and risk of cancer: a systematic review and meta-analysis. Diabetes Care.

[84] Qiao L, Ma D, Lv H (2020). Metabolic syndrome and the incidence of lung cancer: a meta-analysis of cohort studies. Diabetol Metab Syndr..

[85] Wang H, Yan F, Cui Y, Chen F, Wang G, Cui W (2023). Association between triglyceride glucose index and risk of cancer: a meta-analysis. Front Endocrinol (Lausanne)..

