# Supplementary material for: Association between insulin resistance, metabolic syndrome and its components and lung cancer: a systematic review and meta-analysis
Source: Diabetol Metab Syndr. 2024 Mar 11;16:63. doi: 10.1186/s13098-024-01308-w (PMC10926619; doi:10.1186/s13098-024-01308-w)
Supplement: Supplementary file 1 — Additional file 1: Table S1. PRISMA checklist. Table S2. PubMed retrieval strategy. Table S3. Literature characteristics. Figure S1. Analysis figure of metabolic factors unrelated to lung cancer. A TG. B LDL-C. C Hypertension. D FBG. E Obesity. [file 13098_2024_1308_MOESM1_ESM.docx]

***Additional File 1***

**Association between insulin resistance, metabolic syndrome and its components and lung cancer incidence: A systematic review and meta-analysis**

**1. Additional Tables**

**Table S1.** **Preferred Reporting Items for Systematic Reviews and Meta-Analyses (PRISMA)2020 Checklists**

| **Section and Topic** | **Item #** | **Checklist item** | **Location where item is reported** |
| --- | --- | --- | --- |
| **TITLE** | | |  |
| Title | 1 | Identify the report as a systematic review. | Page 1 |
| **ABSTRACT** | | |  |
| Abstract | 2 | See the PRISMA 2020 for Abstracts checklist. |  |
| **INTRODUCTION** | | |  |
| Rationale | 3 | Describe the rationale for the review in the context of existing knowledge. | Page 2-3 |
| Objectives | 4 | Provide an explicit statement of the objective(s) or question(s) the review addresses. | Page 3 |
| **METHODS** | | |  |
| Eligibility criteria | 5 | Specify the inclusion and exclusion criteria for the review and how studies were grouped for the syntheses. | Page 4 |
| Information sources | 6 | Specify all databases, registers, websites, organisations, reference lists and other sources searched or consulted to identify studies. Specify the date when each source was last searched or consulted. | Page 4 |
| Search strategy | 7 | Present the full search strategies for all databases, registers and websites, including any filters and limits used. | Page 4 |
| Selection process | 8 | Specify the methods used to decide whether a study met the inclusion criteria of the review, including how many reviewers screened each record and each report retrieved, whether they worked independently, and if applicable, details of automation tools used in the process. | Page 4 |
| Data collection process | 9 | Specify the methods used to collect data from reports, including how many reviewers collected data from each report, whether they worked independently, any processes for obtaining or confirming data from study investigators, and if applicable, details of automation tools used in the process. | Page 5-6 |
| Data items | 10a | List and define all outcomes for which data were sought. Specify whether all results that were compatible with each outcome domain in each study were sought (e.g. for all measures, time points, analyses), and if not, the methods used to decide which results to collect. | Additional File Table.S3 |
|  | 10b | List and define all other variables for which data were sought (e.g. participant and intervention characteristics, funding sources). Describe any assumptions made about any missing or unclear information. | Additional File Table.S3 |
| Study risk of bias assessment | 11 | Specify the methods used to assess risk of bias in the included studies, including details of the tool(s) used, how many reviewers assessed each study and whether they worked independently, and if applicable, details of automation tools used in the process. | Page 6 |
| Effect measures | 12 | Specify for each outcome the effect measure(s) (e.g. risk ratio, mean difference) used in the synthesis or presentation of results. | Page 5-6 |
| Synthesis methods | 13a | Describe the processes used to decide which studies were eligible for each synthesis (e.g. tabulating the study intervention characteristics and comparing against the planned groups for each synthesis (item #5)). | Page 5-6 |
|  | 13b | Describe any methods required to prepare the data for presentation or synthesis, such as handling of missing summary statistics, or data conversions. | Page 5-6 |
|  | 13c | Describe any methods used to tabulate or visually display results of individual studies and syntheses. | Page 6 |
|  | 13d | Describe any methods used to synthesize results and provide a rationale for the choice(s). If meta-analysis was performed, describe the model(s), method(s) to identify the presence and extent of statistical heterogeneity, and software package(s) used. | Page 6 |
|  | 13e | Describe any methods used to explore possible causes of heterogeneity among study results (e.g. subgroup analysis, meta-regression). | Page 5-6 |
|  | 13f | Describe any sensitivity analyses conducted to assess robustness of the synthesized results. | Page 6 |
| Reporting bias assessment | 14 | Describe any methods used to assess risk of bias due to missing results in a synthesis (arising from reporting biases). | Page 6 |
| Certainty assessment | 15 | Describe any methods used to assess certainty (or confidence) in the body of evidence for an outcome. | Page 6 |
| **RESULTS** | | |  |
| Study selection | 16a | Describe the results of the search and selection process, from the number of records identified in the search to the number of studies included in the review, ideally using a flow diagram. | Page 6-7 |
|  | 16b | Cite studies that might appear to meet the inclusion criteria, but which were excluded, and explain why they were excluded. | Page 7, Fig1 |
| Study characteristics | 17 | Cite each included study and present its characteristics. | Additional File Table.S3 |
| Risk of bias in studies | 18 | Present assessments of risk of bias for each included study. | Additional File Table.S3 |
| Results of individual studies | 19 | For all outcomes, present, for each study: (a) summary statistics for each group (where appropriate) and (b) an effect estimate and its precision (e.g. confidence/credible interval), ideally using structured tables or plots. | Page 8-9, Tab1 |
| Results of syntheses | 20a | For each synthesis, briefly summarise the characteristics and risk of bias among contributing studies. | Page 7-8 |
|  | 20b | Present results of all statistical syntheses conducted. If meta-analysis was done, present for each the summary estimate and its precision (e.g. confidence/credible interval) and measures of statistical heterogeneity. If comparing groups, describe the direction of the effect. | Page 8-9, Tab1 |
|  | 20c | Present results of all investigations of possible causes of heterogeneity among study results. | Page 10-12 |
|  | 20d | Present results of all sensitivity analyses conducted to assess the robustness of the synthesized results. | Page 10-12 |
| Reporting biases | 21 | Present assessments of risk of bias due to missing results (arising from reporting biases) for each synthesis assessed. | Page 13 |
| Certainty of evidence | 22 | Present assessments of certainty (or confidence) in the body of evidence for each outcome assessed. | Page 13 |
| **DISCUSSION** | | |  |
| Discussion | 23a | Provide a general interpretation of the results in the context of other evidence. | Page 14-18 |
|  | 23b | Discuss any limitations of the evidence included in the review. | Page 18 |
|  | 23c | Discuss any limitations of the review processes used. | Page 18 |
|  | 23d | Discuss implications of the results for practice, policy, and future research. | Page 18 |
| **OTHER INFORMATION** | | |  |
| Registration and protocol | 24a | Provide registration information for the review, including register name and registration number, or state that the review was not registered. | Page 1 |
|  | 24b | Indicate where the review protocol can be accessed, or state that a protocol was not prepared. | Page 1 |
|  | 24c | Describe and explain any amendments to information provided at registration or in the protocol. |  |
| Support | 25 | Describe sources of financial or non-financial support for the review, and the role of the funders or sponsors in the review. | Page 21 |
| Competing interests | 26 | Declare any competing interests of review authors. | Page 20-21 |
| Availability of data, code and other materials | 27 | Report which of the following are publicly available and where they can be found: template data collection forms; data extracted from included studies; data used for all analyses; analytic code; any other materials used in the review. | Page 20 |

**PRISMA 2020 Abstract Checklists**

| **Topic** | **No.** | **Item** | **Reported?** |
| --- | --- | --- | --- |
| **TITLE** |  |  |  |
| **Title** | 1 | Identify the report as a systematic review. | Yes |
| **BACKGROUND** |  |  |  |
| **Objectives** | 2 | Provide an explicit statement of the main objective(s) or question(s) the review addresses. | Yes |
| **METHODS** |  |  |  |
| **Eligibility criteria** | 3 | Specify the inclusion and exclusion criteria for the review. | Yes |
| **Information sources** | 4 | Specify the information sources (e.g. databases, registers) used to identify studies and the date when each was last searched. | Yes |
| **Risk of bias** | 5 | Specify the methods used to assess risk of bias in the included studies. | Yes |
| **Synthesis of results** | 6 | Specify the methods used to present and synthesize results. | Yes |
| **RESULTS** |  |  |  |
| **Included studies** | 7 | Give the total number of included studies and participants and summarise relevant characteristics of studies. | Yes |
| **Synthesis of results** | 8 | Present results for main outcomes, preferably indicating the number of included studies and participants for each. If meta-analysis was done, report the summary estimate and confidence/credible interval. If comparing groups, indicate the direction of the effect (i.e. which group is favoured). | Yes |
| **DISCUSSION** |  |  |  |
| **Limitations of evidence** | 9 | Provide a brief summary of the limitations of the evidence included in the review (e.g. study risk of bias, inconsistency and imprecision). | Yes |
| **Interpretation** | 10 | Provide a general interpretation of the results and important implications. | Yes |
| **OTHER** |  |  |  |
| **Funding** | 11 | Specify the primary source of funding for the review. | Yes |
| **Registration** | 12 | Provide the register name and registration number. | Yes |

**Table S2.** **PubMed retrieval strategy**

| Database | PubMed |
| --- | --- |
| Date Searched | 30 June 2023 |
| Search Terms | #1= ((lung cancer[Title/Abstract]) OR (Pulmonary Neoplasm[Title/Abstract])) OR (Lung carcinoma[Title/Abstract])  #2= (((((((hyperlipidemia[Title/Abstract]) OR (obesity[Title/Abstract])) OR (dyslipidemia[Title/Abstract])) OR (hypertension[Title/Abstract])) OR (hyperglycemia[Title/Abstract])) OR (insulin resistance[Title/Abstract])) OR (metabolic syndrome[Title/Abstract])) OR (diabetes[Title/Abstract])  #3= ((cohort[Title/Abstract]) OR (case-control[Title/Abstract])) OR (cross-sectional[Title/Abstract])  #4= ("1855/01/01"[Date - Publication]: "2023/06/30"[Date - Publication])  #5= #1 AND #2 AND #3 AND #4 |
| Results Returned | 511 |

**Table S3.** Literature characteristics

| Author | Year | Location, follow-up | Number of cases/participants | Exposure details | Adjusted RR (95% CI) | Quality | Comparison | Adjustments |
| --- | --- | --- | --- | --- | --- | --- | --- | --- |
| [Tomàs López Jiménez](https://pubmed.ncbi.nlm.nih.gov/?term=L%C3%B3pez-Jim%C3%A9nez+T&cauthor_id=35245317) et al ^[12]^ | 2022 | Catalonia  2006-2017 | 20,387/916,420 | MetS(M)  MetS(W) | 1.14(1.08-2.20)  1.01(0.90-1.12) | 7 | NCEP ATP III | age, nationality, smoking status and MEDEA Deprivation Index |
| [Yoneatsu Osaki](https://pubmed.ncbi.nlm.nih.gov/?term=Osaki+Y&cauthor_id=21890443) et al ^[13]^ | 2012 | Japan  1992-2000 | 211/23625 | MetS(M)  MetS(W) | 0.54(0.27-1.11)  0.99(0.52-1.87) | 8 | NCEP ATP III | age, consumption of large quantities of alcohol and smoking status |
| Manami Inoue et al ^[30]^ | 2006 | Japan  1990-1994 | 745/97,771 | DM(M)  DM(W) | 1.05 (0.77-1.44)  1.12(0.55-2.29) | 8 | Yes vs. No | Baseline age, smoking, BMI, study area, history of cerebrovascular disease, history of ischaemic heart disease, ethanol intake, leisure time physical activity, green vegetable intake and coffee intake. |
| K. Rapp et al ^[15]^ | 2006 | Austria  1988-2001 | 105/155,820 | DM(M) | 1.40(0.95-2.06) | 6 | Yes vs. No | Smoking, BMI and occupational group. |
| Kiyonori Kuriki et al ^[16]^ | 2007 | Japan  1988-2000 | 1,520/59,440 | DM(M)  DM(W) | 1.53(1.22–1.93)  1.61(1.01–2.55) | 7 | Yes vs. No | Age, BMI, drinking and smoking habits, bowel movement, family history of cancer for each specific site, family history of DM, regular physical exercise, dietary restriction, raw vegetable intake, greasy food intake and snacking. |
| Johnson JA et al ^[17]^ | 2011 | Canada  1994-2006 | 3,211/370,200 | DM | 1.14(1.06-1.24) | 8 | Yes vs. No | Age, sex, family-level socioeconomic status, frequency of physician visits in the 2 years prior to the index date and index year. |
| Chin-Hsiao Tseng et al ^[42]^ | 2014 | Taiwan,China  1979-2007 | 771/996,950 | Hypertension  Obesity | 0.99(0.82-1.20)  1.11(0.53-2.35) | 7 | Yes vs. No  Yes vs. No | Age, occupation, living region, chest X-ray examination, drugs, comorbidities, DM duration, hypertension, chronic obstructive pulmonary disease, stroke, nephropathy, ischemic heart disease, peripheral arterial disease, eye disease, obesity, dyslipidemia. |
| Hyung Jun Park et al ^[18]^ | 2019 | Korea  2005-2013 | 1,972/300,518 | DM | 0.91(0.71-1.17) | 8 | Yes vs. No | Age, sex, household income, alcohol consumption, physical activity, BMI, T2DM, systolic blood pressure, serum TC, charlson comorbidity index. |
| Wan-Shui Yang et al ^[19]^ | 2014 | China  1996-2000 | 1,017/133,024 | DM(M)  DM(W) | 0.87(0.62-1.21)  0.93(0.69-1.25) | 8 | Yes vs. No | Age, BMI, birth cohort, income, education, occupation, smoking status, alcohol drinking status, family history of cancer, total energy intake, fruit intake, vegetable intake, total physical activity, history of hepatitis/chronic liver disease, hormone replacement therap, menopausal status. |
| Xiang Li ^[28]^ | 2012 | China  2005-2010 | 96/336 | DM  BMI  TG  TC  LDL-C  HDL-C | 0.88(0.53-1.45)  0.63(0.39-1.02)  0.82(0.56-1.20)  0.59(0.41-0.84)  0.95(0.65-1.40)  1.11(0.64-1.93) | 6 | >7 vs ≤7 mmol/L  ≥24 vs <24kg/m^2^  ≥0.8 vs <0.8mmol/L  ≥4.3 vs <4.3mmol/L  ≥2.8 vs <2.8mmol/L  ≥0.9 vs <0.9mmol/L | - |
| Xu Wang et al ^[20]^ | 2016 | China  2010-2015 | 34/134 | DM | 0.57(0.21-1.58) | 5 | Yes vs. No | - |
| Amanda Leiter et al ^[21]^ | 2021 | The United States  1993-2001 | 3,449/140,935 | DM | 1.03(0.91-1.17) | 7 | Yes vs. No | Age, sex, BMI, education, race/ethnicity, smoking, family history, personal history, chronic obstructive pulmonary disease. |
| Sun Ha Jee et al ^[22]^ | 2005 | Korea  1993-2002 | 853/1,298,385 | DM(M)  DM(W)  FBG(M)  FBG(W) | 1.06(0.96-1.16) 1.16(0.94-1.44)  1.01(0.90-1.14)  1.13(0.85-1.51) | 8 | Yes vs. No  Q5 vs Q1(≥140 vs <90mg/dL) | Age, smoking and alcohol use. |
| Pär Stattin et al ^[23]^ | 2007 | Northern Sweden  1989-2002 | 114/64,597 | DM | 1.40(0.85-2.38) | 5 | Yes vs. No | Smoking status, BMI, age, year of recruitment, fasting time. |
| Juhua Luo et al ^[24]^ | 2012 | The United States  1993-2010 | 2,257/145,765 | DM | 1.09(0.89-1.33) | 8 | Yes vs. No | Age, BMI, waist-to-hip ratio, ethnicity, education, alcohol consumption, smoking status, physical activity, total energy intake, percent calories from fat, total fruit intake and total vegetable intake, history of hormone therapy use, and different treatment assignments in clinical trials. |
| Gabriel Chodick et al ^[25]^ | 2010 | Israel  2000-2008 | 267/100,595 | DM(M)  DM(W) | 1.76 (1.17-2.64)  0.85 (0.43-1.68) | 8 | Yes vs. No | Age, sex, BMI, smoking history, marital status, place of residence, severity of DM, years of stay in Israel, and socioeconomic level defined according to the poverty index based on patient's residence. |
| Tanja Stocks et al ^[26]^ | 2009 | Norway, Austria, and Sweden  1992-2006 | 2,953/549,944 | DM(M)  DM(W) | 1.42(0.90-2.16)  1.25(0.54-2.93) | 8 | Yes vs. No | Age, smoking status, BMI and analyses of glucose. |
| Bo Hao et al ^[27]^ | 2018 | China  2016-2018 | 424/838 | DM  BMI  TC  TG  LDL-C  HDL-C | 1.18(0.55-2.49)  0.71(0.50-1.00)  1.49(0.88-2.52)  1.05(0.67-1.65)  0.20(0.03-1.36)  0.23(0.13-0.41) | 7 | Yes vs. No  >25 vs ≤25g/cm^2^  ≥6.2 vs <5.2mmol/L  ≥2.3 vs <1.7mmol/L  ≥4.1 vs <3.4 mmol/L  ≥1.5 vs <1.0mmol/L | Age, sex, BMI, smoking status, hypertension, DM and lipid profiles |
| Zhangyan Lyu ^[29]^ | 2019 | China  2006-2015 | 986/109,798 | TC  TG  LDL-C  HDL-C | 1.30(1.04-1.63)  1.27(1.01-1.59)  1.19(0.94-1.51)  0.93(0.75-1.15) | 7 | ≥220 vs <160mg/dl  ≥200 vs <75mg/dL  ≥120 vs <70mg/dL  ≥69 vs <46mg/dL | Age, BMI, smoking status, alcohol consumption status, educational level, income level, coal dust exposure status, degree of coal dust exposure, physical activity, dietary fat intake, FBG. |
| Manami Inoue et al ^[14]^ | 2009 | Japan  1990-2004 | 224/27,724 | MetS(M)  MetS(W)  Hypertension(M)  Hypertension(W)  Obesity(M)  Obesity(W)  TG(M)  TG(W) | 0.86(0.57-1.30)  0.66(0.35-1.24)  1.17(0.81-1.68)  0.75(0.47-1.21)  1.10(0.76-1.60)  0.71(0.42-1.20)  0.93(0.63-1.38)  0.98(0.57-1.70) | 9 | NCEP ATP III  ≥130/85 vs <130/85mmHg  ≥25 vs <25kg/m^2^  ≥1.69 vs <1.69mmol/l | Age at baseline, smoking status, ethanol intake status, daily total physical activity level, TC and study area. |
| Jiyoung Ahn et al ^[31]^ | 2009 | Finland  1985-2003 | 2,618/29,093 | TC  HDL-C | 0.81(0.72-0.92)  0.89(0.78-1.01) | 8 | Q5 vs Q1(>276.7 vs <203.9mg/dL)  Q5 vs Q1(>55.3 vs <36.2mg/dL) | Age, BMI, level of education, systolic blood pressure, physical activity, smoking, alcohol consumption, saturated fat intake, polyunsaturated fat intake, intervention, serum TC, and serum HDL-C. |
| Cari M. Kitahara et al ^[32]^ | 2011 | Korea  1992-2006 | 10,866/1,189,719 | TC(M)  TC(W) | 0.89(0.82-0.96)  0.91(0.78-1.07) | 8 | ≥240 vs <160mg/dL | BMI, smoking, alcohol drinking, fasting serum glucose, hypertension and physical activity. |
| Anna M Kucharska-Newton et al ^[33]^ | 2008 | The United States  1987-2000 | 259/14,547 | HDL-C | 0.69(0.52-0.91) | 6 | Q4 vs Q1  (M≥51.0 vs <34.7mg/dl,W≥67.4 vs <45.3 mg/dl) | Age, sex, BMI, race, alcohol consumption, cigarette pack-years of smoking, TG and exercise. |
| Chao Zhao et al ^[34]^ | 2021 | China  2017-2019 | 1,593/3,186 | TG  TC  LDL-C | 0.89(0.74-1.06)  0.63(0.53-0.76) 0.92(0.75-1.13) | 5 | ≥1.7 vs <1.7mmol/l  ≥5.17 vs <5.17mmol/l  ≥3.33 vs <3.33mmol/l | Age, sex, smoking, alcohol consumption, other lipid composition, etc. |
| Seulki Ko et al ^[35]^ | 2016 | Korea  2002-2013 | 624/99,565 | TC(M)  TC(W)  FBG(M)  FBG(W)  Hypertension(M)  Hypertension(W)  Obesity(M)  Obesity(W) | 1.21(0.87-1.70)  0.87(0.49-1.57)  1.22(0.89-1.67)  0.76(0.44-1.32)  1.05(0.64-1.73)  0.93(0.43-2.01)  1.29(0.94-1.77)  1.19(0.71-1.19) | 8 | ≥200 vs <200 mg/dL  ≥100 vs <100mg/dL  ≥130/85 vs <130/85mmHg  ≥25 vs <25kg/m^2^ | Age, smoking consumption, regular exercise and alcohol intake. |
| Susanne Strohmaier et al ^[36]^ | 2013 | Norway, Austria, and Sweden  1972-2006 | 3,869/577,330 | TC(M)  TC(W) | 1.15(0.95-1.40) 1.24(0.88-1.76) | 7 | Q5 vs Q1 | Age, BMI, and smoking status. |
| Rūta Everatt et al ^[37]^ | 2014 | Lithuania  1978-2008 | 358/6,729 | TC  BMI | 0.75(0.53-1.05)  0.69(0.49-0.97) | 7 | Q5 vs Q1  (>265 vs <194mg/dL)  ≥30 vs <25 kg/m^2^ | Age, smoking and alcohol consumption, BMI, type of cigarettes, education. |
| Eleni Th. Petridou et al ^[38]^ | 2011 | Athens  2002-2005 | 81/243 | IR  BMI | 2.02(0.88-4.65)  0.49(0.27-0.90) | 7 | Q5 vs Q1  Q5 vs Q1 | HOMA-IR, BMI, weight change, waist-to-hip ratio, smoking, alcohol, education, leptin, adiponectin, and coffee consumption |
| Ilona Argirion et al ^[39]^ | 2017 | Finland  1985-2003 | 209/591 | FBG  IR | 0.68(0.38-1.20)  1.83(0.99-3.38) | 7 | Q4 vs Q1(>107 vs ≤92mg/dL)  Q4 vs Q1(>1.53 vs ≤0.67) | Age, BMI, smoking and family history |
| Bing Bai ^[40]^ | 2016 | China  2013-2015 | 92/179 | IR | 4.48(1.71-11.73) | 6 | - | Age, sex, BMI, etc |
| Wenfei Zhao ^[41]^ | 2017 | China  2013-2014 | 81/162 | IR | 3.48(0.85-14.27) | 5 | - | - |

**Abbreviations:** MetS: metabolic syndrome, DM: diabetes mellitus, FBG: fasting blood glucose, TG: triglyceride, TC: total cholesterol, HDL-C: high-density lipid-cholesterol, LDL-C: low-density lipoprotein cholesterol, BMI: body mass index, IR: insulin resistance

**2.** **Additional Figures**

**Figure S1. Analysis figure of metabolic factors unrelated to lung cancer.**


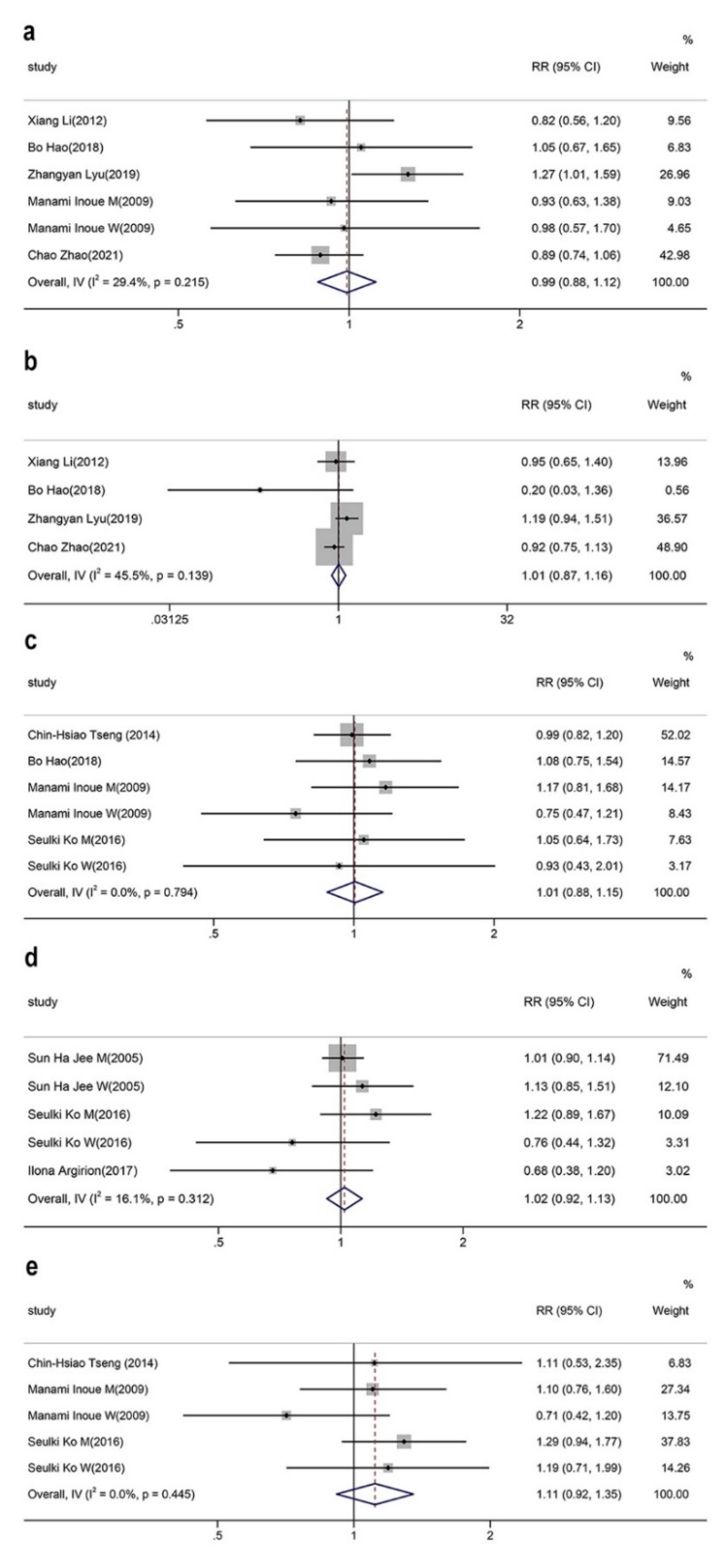


A, TG. B, LDL-C. C, Hypertension. D, FBG. E, Obesity.
